# Neutrophil-to-Lymphocyte Ratio, Platelet-to-Lymphocyte Ratio, and Monocyte-to-Lymphocyte Ratio in Depression: An Updated Systematic Review and Meta-Analysis

**DOI:** 10.3389/fpsyt.2022.893097

**Published:** 2022-06-15

**Authors:** Yanwei Cheng, Yiwen Wang, Xiangyi Wang, Zhuoya Jiang, Lijun Zhu, Shaokuan Fang

**Affiliations:** ^1^Department of Neurology, Neuroscience Centre, The First Hospital of Jilin University, Changchun, China; ^2^China-Japan Union Hospital of Jilin University, Changchun, China

**Keywords:** meta-analysis, depression, neutrophil-to-lymphocyte ratio, platelet-to-lymphocyte ratio, monocyte-to-lymphocyte ratio

## Abstract

**Background:**

Research on neutrophil-to-lymphocyte ratio (NLR), platelet-to-lymphocyte ratio (PLR), and monocyte-to-lymphocyte ratio (MLR) in depression is still emerging and has increased 3-fold since the first meta-analysis. An updated meta-analysis with sufficient studies can provide more evidence for a potential relationship between NLR, PLR, MLR, and depression.

**Methods:**

We identified 18 studies from the PubMed, EMBASE, Cochrane library, and Web of Science databases. Meta-analyses were performed to generate pooled standardized mean differences (SMDs) and 95% confidence intervals (CIs) between patients with depression and controls. Sensitivity analysis, subgroup analysis, meta-regression, and publication bias were conducted.

**Results:**

A total of 18 studies including 2,264 depressed patients and 2,415 controls were included. Depressed patients had significantly higher NLR and PLR compared with controls (SMD = 0.33, 95% CI: 0.15–0.52, *p* < 0.001 and SMD = 0.24, 95% CI: 0.02–0.46, *p* < 0.05, respectively). MLR was slightly higher in depressed individuals compared to controls (SMD = 0.15, 95% CI: −0.26 to 0.55, *p* > 0.05), despite the absence of significance. Sensitivity analysis removing one study responsible for heterogeneity showed a higher and significant effect (SMD = 0.32, 95% CI: 0.20–0.44) of MLR. Three subgroup analyses of NLR, PLR, MLR, and depression revealed obvious differences in the inflammatory ratios between depressed patients and controls in China and the matched age and gender subgroup. Individuals with post-stroke depression (PSD) had higher NLR and MLR values as compared to non-PSD patients (SMD = 0.51, 95% CI: 0.36–0.67, *p* < 0.001 and SMD = 0.46, 95% CI: 0.12–0.79, *p* < 0.01, respectively). Meta-regression analyses showed that male proportion in the case group influenced the heterogeneity among studies that measured NLR values (*p* < 0.05).

**Conclusions:**

Higher inflammatory ratios, especially NLR, were significantly associated with an increased risk of depression. In the subgroup of China and matched age and gender, NLR, PLR, and MLR were all elevated in depressed patients vs. controls. Individuals with PSD had higher NLR and MLR values as compared to non-PSD patients. Gender differences may have an effect on NLR values in patients with depression.

## Introduction

Major depressive disorder (MDD) is a common and severe mental illness characterized by prominent and persistent low mood, cognitive impairment, and impaired social function (1). MDD has a significant impact on a person's quality of life at work, at school, and in their family and is also a major cause of death by suicide, which is often ignored. MDD affects more than 300 million people worldwide and causes severe social and economic burden (2). Genetic factors confer some heritable risk of MDD, while structural and neurochemical deficits in brain regions and exposure to traumatic or repeated psychosocial and environmental stressors can increase vulnerability to MDD or induce depressive symptoms (3, 4). Depression is a multifactorial and heterogeneous disease with overlapping causal pathways, its pathogenesis has not been completely elucidated so far. The current clinical diagnosis of depression depends on symptoms of patients and depression rating scales, which is high subjectivity and misdiagnosis rate, and lacks reliable biomarkers.

There is increasing evidence to suggest that the immune and inflammatory system plays a role in the pathophysiology of depression (4–6). Individuals with MDD have been shown to have increased numbers of circulating granulocytes and monocytes, elevated levels of circulating pro-inflammatory cytokines and acute phase proteins (7–9). Furthermore, a previous study suggested that elevated levels of peripheral inflammatory markers in patients with MDD were associated with increased anhedonia (10). Peripheral inflammation can be transmitted to the brain where it is integrated by microglia, which collaboratively contributes to development of neuroinflammation in depression. Studies on MDD have revealed that robust changes in microglial morphology and density, which are characteristic of neuroinflammation, were observed in the prefrontal cortex and hippocampus of post-mortem brains (11, 12). Meanwhile, a neuroimaging study showed that translocator protein density, a marker of microglial activation, was elevated in brain regions of individuals with major depressive episode (13). Furthermore, some researchers have found that patients treated with cytokines for various illnesses were at increased risk of developing depressive symptoms (14–16). Taken together, immune dysregulation of the peripheral and central immune system is involved in the pathophyisiology of MDD.

Neutrophil-to-lymphocyte ratio (NLR), platelet-to-lymphocyte ratio (PLR), and monocyte-to-lymphocyte ratio (MLR) are inexpensive and easily acquired reproducible markers of systemic inflammation (17–19), and are calculated from white blood cell counts. These inflammatory ratios could have better predictive value in evaluating inflammation than neutrophils, platelets, monocytes, or lymphocytes separately due to the lower effect of confounding conditions (20). It has been found that they can be utilized as biomarkers of inflammation or poor prognosis in various diseases, such as cancers (21–23), cerebrovascular diseases (24, 25), cardiovascular diseases (26, 27), and autoimmune diseases (28–31). In recent years, a growing number of studies have investigated NLR, PLR, and MLR in psychological disorders, and showed that they were higher when compared to healthy controls (32–36). NLR, PLR, and MLR were all found to be elevated in patients with depression (37–41). Moreover, several studies have suggested that NLR was positively associated with severity of depression and might be a peripheral marker for suicidal vulnerability and behavior in depressed patients (42, 43, 103). Additional studies also pointed out that PLR seemed to be more predictive in evaluating the prognosis of major depression (44) and MLR was positively correlated with suicidal attempts in adolescents with depression (105).

A previous meta-analysis reported that patients with MDD had higher NLR compared with healthy controls (45). However, the number of studies selected in the meta-analysis was relatively low. As the number of studies on the association between NLR, PLR, MLR, and depression is increasing, different voices have emerged (46–49). Therefore, an updated meta-analysis up to August 2021 was performed. We aimed to provide reliable evidence regarding the role of these inflammation ratios in depression to inform future studies.

## Methods

We performed this systematic review and meta-analysis based on the Preferred Reporting Items for Systematic Reviews and Meta-analysis (PRISMA) standard (50).

### Search Strategy

We conducted a comprehensive literature search in the PubMed, EMBASE, Cochrane library, and Web of Science electronic databases. The following search strategy was used: (“neutrophil-to-lymphocyte ratio” OR “NLR” OR “neutrophil to lymphocyte ratio” OR “neutrophil/lymphocyte ratio” OR “neutrophil-lymphocyte ratio” OR “neutrophil lymphocyte ratio”) AND (“platelet-to-lymphocyte ratio” OR “PLR” OR “platelet to lymphocyte ratio” OR “platelet/lymphocyte ratio” OR “platelet-lymphocyte ratio” OR “platelet lymphocyte ratio”) AND (“monocyte-to-lymphocyte ratio” OR “MLR” OR “monocyte to lymphocyte ratio” OR “monocyte/lymphocyte ratio” OR “monocyte-lymphocyte ratio” OR “monocyte lymphocyte ratio”) AND (“depression” OR “affective” OR “mood” OR “depressive disorder” “major depression” OR “major depressive disorder” OR “MDD”). The search period was from the creation of the databases up until August 2021. We also screened references from selected articles to confirm potentially related studies.

### Inclusion and Exclusion Criteria

Studies were included in this meta-analysis if they satisfied the following criteria: (1) study design: observational studies (cohort, case-control, or cross-sectional); (2) participants: patients who were clearly diagnosed as depressed according to the Diagnostic and Statistical Manual of Mental Disorders (DSM) or International Classification of Diseases (ICD), and controls without mental disorders; (3) comparative data on NLR, MLR, and PLR between cases and controls could be extracted. The exclusion criteria were: (1) duplicate data; (2) reviews, letters, conference abstracts, or meta-analyses; (3) non-human studies and mechanism studies; (4) non-English publications; and (5) non-adult participants. Furthermore, the latest or the most complete study was selected when participants overlapped in several studies. Two researchers (Yanwei Cheng and Yiwen Wang) worked independently to complete the preliminary screening through browsing titles and abstracts, and made final decisions after reviewing the full texts. Disagreements between the researchers were resolved by group discussions.

### Data Extraction

Two investigators (Yanwei Cheng and Xiangyi Wang) extracted original data from the selected studies. The following information was collected: surname of the first author, publication year, country, type of depression, diagnostic criteria, sample size, mean age, percentage of men, and peripheral blood cell ratios. When the data on peripheral blood cell ratio were expressed as being in the median and interquartile range (IQR), we referenced the method of McGrath et al. (51) to estimate the mean and standard deviation (SD). Any divergences were figured out through discussion between the two investigators.

### Quality Assessment

The quality of the selected investigations was independently evaluated by two authors (Yanwei Cheng and Zhuoya Jiang) with the Newcastle-Ottawa scale (NOS). We assessed a study with respect to three aspects: Selection, Comparability, and Exposure or Outcome. A maximum of one star was awarded in each category for Selection and Exposure or Outcome, while a maximum of two stars could be awarded for Comparability. The total score ranged from 0 to 9, and a study could be qualified as high-quality when the total score was not <5. Disagreements were managed by group discussion.

### Statistical Analysis

The meta-analysis was carried out using STATA 12.0 statistical software. Standardized mean differences (SMDs) and 95% confidence intervals (CIs) were used to construct forest plots of continuous data and to estimate the association of NLR, PLR, and MLR with depression susceptibility. A *p*-value < 0.05 was considered statistically significant for the pooled SMDs measured by *Z*-test. Heterogeneity across studies was measured based on the *p*-value of the chi-square and *I*^2^-statistic. Heterogeneity was considered high if the *p*-value was < 0.10 or *I*^2^ > 50%, and random-effects models were used. A fixed-effects model was applied when the *p*-value was ≥ 0.10 or *I*^2^ ≤ 50%. We conducted a sensitivity analysis in order to investigate the influence of a single study on the overall result, and to search for whether a study is the source of heterogeneity. Subgroup analysis was carried out to seek the potential source of heterogeneity, and to explore whether the estimated effect could vary in the following aspects, country of origin (China, Turkey, or other countries), type of depression [MDD, post-stroke depression (PSD)], diagnostic criteria (DSM or ICD), matching age and sex simultaneously (Yes vs. No), suicidal behavior including suicide attempt and suicidal ideation (Yes vs. No). Further, we performed meta-regression analyses to identify potential sources of heterogeneity by adjusting mean age and the male proportion. In addition, publication bias was assessed by the funnel plot, Begg's test, and Egger's test.

## Results

### Study Selection

**Figure 1:**
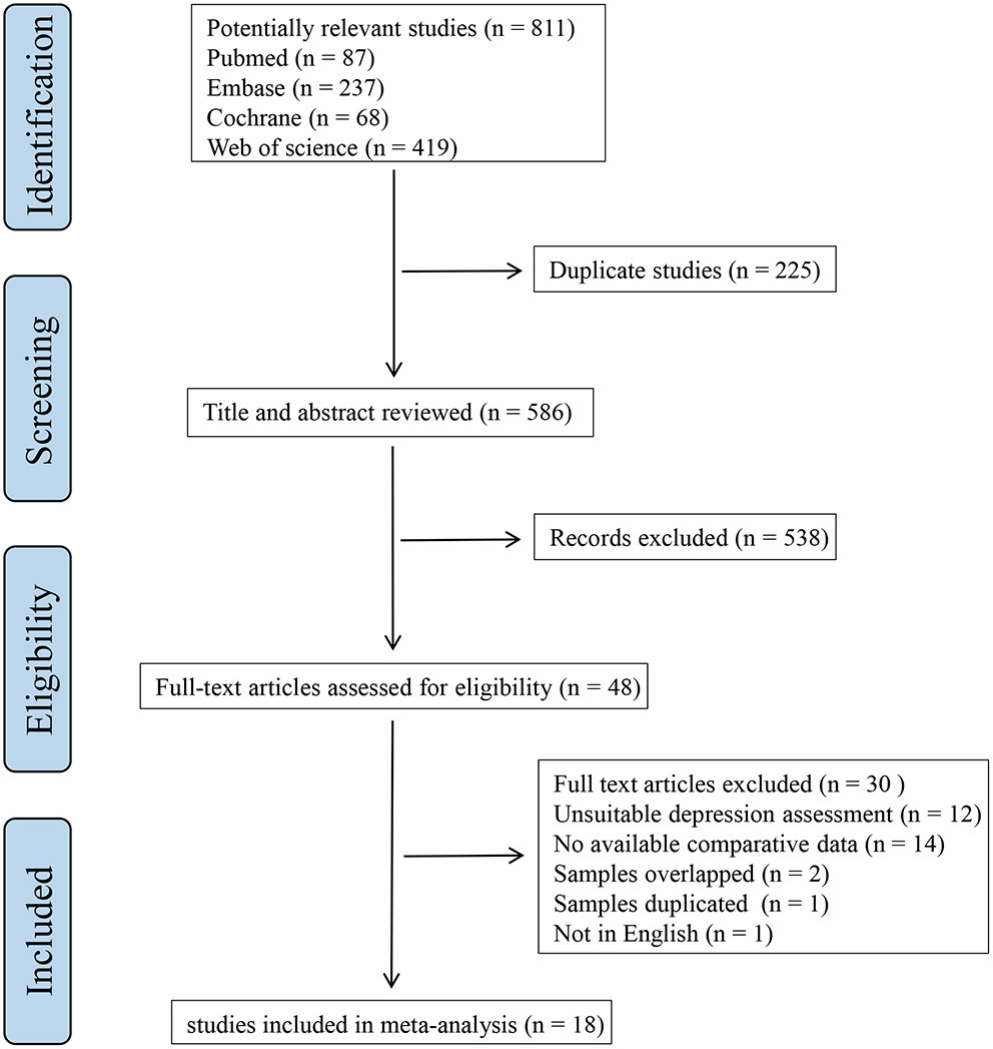
Flowchart of literature search and selection.

A flowchart representing the screening procedure is shown in Figure 1. A total of 811 articles were identified after the initial search. We removed 225 duplicates and 538 studies were then excluded by browsing the titles and abstracts. After a full-text review of 48 articles, we eventually selected 18 research that met the eligibility criteria.

### Characteristics and Quality Assessment of Studies

The eighteen studies (17, 35, 37, 38, 41, 42, 46–49, 52–56, 102) selected in this meta-analysis were published between 2015 and 2021, and consisted of 2,264 depression cases and 2,415 controls. As displayed in Table 1, we summarized the main characteristics of these studies. Geographically, seven investigations were implemented in China, seven in Turkey, one in Spain, one in the USA, one in Poland, and one in Germany. These studies diagnosed different types of depression, PSD in three, and MDD in 15. Patients with depression were diagnosed using the DSM-IV, DSM-5, and ICD-10 scales, and 15 studies had age- and sex-matched controls while the controls of the remaining three studies did not match the cases in age or gender. For suicidal behavior, only three studies have classified patients with depression into those with and those without suicide attempt or suicidal ideation. The association between NLR, PLR, MLR and susceptibility to depression were evaluated in 17 studies, 10 studies, and three studies, respectively. The NLR, PLR, and MLR values of each study are presented in Table 2. The selected studies displayed satisfactory quality with NOS scores of 5–8 points (Supplementary Table S1).

**Table 1:** Main characteristics of studies included in the meta-analysis.

**References**	**Country**	**Diagnosis**	**Diagnostic criteria**	**Matching age and sex simultaneously**	**Individuals with depression**				**Controls**		**Inflammatory ratio**
					***N***	**Mean age**	**Males (** ***n*** **)**	***N***	**Mean age**	**Males (** ***n*** **)**	
Hu et al. (41)	China	PSD	DSM-IV, HAMD-17 >7	Yes	129	62.0 ± 10.9	75	294	62.8 ± 10.0	197	NLR, PLR
Ding et al. (40)	China	PSD	DSM-IV, HAMD-17 >7	Yes	44	68.11 ± 8.73	30	159	66.16 ± 11.15	108	MLR
Bulut et al. (37)	Turkey	MDD	DSM-V	Yes	93	41.37 ± 13.69	40	95	39.81 ± 12.96	40	NLR, PLR
Zhou et al. (56)	China	MDD	DSM-IV	Yes	454	53.08 ± 9.20	129	458	53.02 ± 6.75	147	NLR, PLR, MLR
Yu et al. (35)	China	MDD	DSM-V	Yes	82	21.06 ± 11.14	36	120	22.63 ± 8.87	54	NLR, PLR, MLR
Sahin et al. (49)	Turkey	MDD	DSM-V, HAMD >7	Yes	57	39.79 ± 13.26	27	59	41.54 ± 15.48	22	NLR, PLR
Martínez-Botía et al. (48)	Spain	MDD	DSM-V	No	71	52.90 ± 10.46	32	93	48.22 ± 11.44	53	NLR, PLR, MLR
Hu et al. (54)	China	PSD	DSM-IV	No	104	61.74 ± 10.52	53	272	60.64 ± 10.02	171	NLR PLR
Grudet et al. (46)	USA	MDD	DSM-IV, HAMD-17 ≥17	Yes	48	39.3 ± 14.9	21	54	37.9 ± 13.9	21	NLR
Öztürk et al. (102)	Turkey	MDD	DSM-IV	Yes	49	40.86 ± 14.7	13	48	36.85 ± 10.1	12	NLR
Arabska et al. (52)	Poland	MDD	ICD-10	No	465	74.8 ± 7.9	110	219	71.1 ± 5.6	48	NLR
Euteneuer et al. (53)	Germany	MDD	DSM-IV	Yes	98	37.3 ± 12.2	50	30	37.1 ± 12.2	15	NLR
Ekinci and Ekinci (42)	Turkey	MDD	DSM-IV	Yes	139	42.18 ± 12.21	42	50	44.12 ± 4.23	13	NLR, PLR
Cai et al. (17)	China	MDD	DSM-IV, HAMD ≥10	Yes	103	46.58 ± 13.38	46	106	46.56 ± 12.94	47	NLR, PLR
Peng et al. (55)	China	MDD	DSM-IV	Yes	167	38.2 ± 9.56	54	180	39.1 ± 10.64	67	NLR, PLR
Korkmaz et al. (47)	Turkey	MDD	DSM-IV	Yes	40	43 ± 12	17	40	43 ± 8	18	NLR
Demircan et al. (39)	Turkey	MDD	DSM-IV	Yes	80	44.10 ± 10.60	44	91	39.80 ± 11.50	49	NLR
Demir et al. (38)	Turkey	MDD	DSM-V	Yes	41	28.4 ± 9.2	11	47	30.0 ± 9.2	12	NLR

*PSD, post-stroke depression; MDD, major depressive disorder; DSM, Diagnostic and Statistical Manual of Mental Disorders; ICD, International Classification of Diseases; NLR, neutrophil-to-lymphocyte ratio; PLR, platelet-to-lymphocyte ratio; MLR, monocyte-to-lymphocyte ratio*.

**Table 2:** Comparison of NLR, PLR, MLR between patients with depression and controls.

**Inflammatory ratio**	**References**	**Patients with depression**		**Controls**	
NLR	Hu et al. (41)^*^	3.01 ± 2.01	129	2.21 ± 0.88	294
	Bulut et al. (37)	2.25 ± 1.50	93	1.67 ± 0.78	95
	Zhou et al. (56)	2.09 ± 1.45	454	2.00 ± 0.73	458
	Yu et al. (35)	1.99 ± 1.18	82	1.78 ± 0.55	120
	Sahin et al. (49)	2.60 ± 1.10	57	1.90 ± 0.62	59
	Martínez-Botía et al. (48)	2.01 ± 1.42	71	1.87 ± 0.80	93
	Hu et al. (54)^*^	3.67 ± 1.59	104	3.13 ± 1.23	272
	Grudet et al. (46)	2.2 ± 0.9	48	2.3 ± 1.2	54
	Öztürk et al. (102)	2.29 ± 1.02	49	2.09 ± 0.95	48
	Arabska et al. (52)	1.78 ± 1.31	465	1.95 ± 0.73	219
	Euteneuer et al. (53)	2.0 ± 0.7	98	1.6 ± 0.6	30
	Ekinci and Ekinci (42)	2.12 ± 0.95	139	1.81 ± 0.33	50
	Cai et al. (17)	2.28 ± 0.62	103	2.0 ± 0.73	106
	Peng et al. (55)	1.9 ± 0.73	167	1.7 ± 0.62	180
	Korkmaz et al. (47)	1.58 ± 0.59	40	2.05 ± 0.89	40
	Demircan et al. (39)	2.55 ± 0.70	80	1.41 ± 0.8	91
	Demir et al. (38)	2.3 ± 0.9	41	2.0 ± 0.6	47
PLR	Hu et al. (41)^*^	125.02 ± 44.40	129	118.92 ± 40.22	294
	Bulut et al. (37)	128.78 ± 59.63	93	110.67 ± 41.86	95
	Zhou et al. (56)	127.86 ± 52.45	454	117.20 ± 36.97	458
	Yu et al. (35)	134.28 ± 47.09	82	113.12 ± 22.57	120
	(49)	122.67 ± 43.92	57	141.11 ± 48.32	59
	(48)	119.49 ± 55.27	71	117.99 ± 54.77	93
	Hu et al. (54)^*^	195.25 ± 112.26	104	120.19 ± 44.15	272
	Ekinci and Ekinci (42)	131.65 ± 59.86	139	134.3 ± 61.4	50
	Cai et al. (17)	128.69 ± 29.73	103	118.79 ± 32.84	106
	Peng et al. (55)	125.3 ± 37.99	167	124.4 ± 59.15	180
MLR	Ding et al. (40)^*^	0.28 ± 0.08	44	0.24 ± 0.09	159
	Zhou et al. (56)	0.18 ± 0.08	454	0.16 ± 0.05	458
	Martínez-Botía et al. (48)	0.26 ± 0.11	71	0.30 ± 0.13	93

*NLR, neutrophil-to-lymphocyte ratio; PLR, platelet-to-lymphocyte ratio; MLR, monocyte-to-lymphocyte ratio*.

* *Mean and standard deviation are estimated from median and interquartile range according to McGrath et al.'s method*.

### Patients With Depression vs. Controls

#### NLR

**Figure 2:**
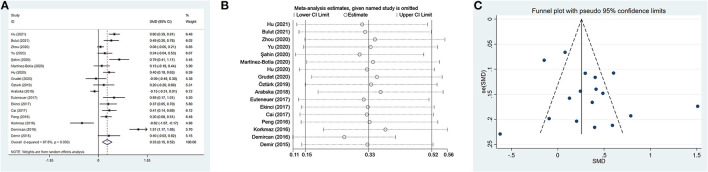
Results of meta-analysis on NLR between depressed patients and controls. **(A)** Forest plot of standardized mean difference in NLR between depressed patients and controls. Depressed patients showed significantly higher NLR values than controls (SMD = 0.33, 95% CI: 0.15–0.52, *p* < 0.001). The random-effects model was used due to substantial heterogeneity across studies (*I*^2^ = 87.6%, *p* < 0.001). **(B)** Sensitivity analysis revealed that the SMD values were relatively stable in the direction and significance of the results. **(C)** The funnel plot was roughly symmetric, which indicated the absence of publication bias in studies evaluating NLR values in depression.

Seventeen studies (17, 35, 37, 38, 41, 42, 46–49, 52–56, 102) including a total of 2,220 patients with depression (800 males) and 2,256 controls (986 males) were evaluated in the meta-analysis. Depressed patients showed significantly higher NLR values compared with controls (SMD = 0.33, 95% CI: 0.15–0.52, *p* < 0.001) (Figure 2A). The random-effects model was used due to substantial heterogeneity across studies (*I*^2^ = 87.6%, *p* < 0.001). Sensitivity analysis revealed that the corresponding SMD values did not obviously change after sequential exclusion of each study, both in terms of direction and significance of the results (Figure 2B).

The results of subgroup analysis based on clinical characteristics (country, type of depression, diagnostic criteria, matching in terms of age and sex, and suicidal behavior) are presented in Table 3. Significant differences in depressed individuals with controls were observed in China and Turkey, but not in other countries (Spain, the USA, Poland, and Germany). The subgroup analysis according to type of depression showed that NLR values were higher in patients with MDD and PSD than in controls. Depressed patients diagnosed based on the DSM had obvious higher NLR values than controls. However, the study by Arabska et al. (52) revealed that NLR values were lower in individuals with depression according to the ICD-10, although the difference was insignificant. In the next subgroup analysis, NLR was significantly elevated in the subgroup of matched age and sex, but not in the unmatched subgroup. Finally, patients with suicidal behavior showed an elevated trend of NLR compared with controls. Whereas, depressed patients without suicidal behavior had slightly lower NLR values than controls, although it was not a statistically significant difference.

**Table 3:** Summary of meta-analyses.

**Peripheral blood cell ratio**	**Included studies**	**Model**	**SMD**	**95%CI**	***I*^2^ (%)**	**P_H_**	**Z**	**P**
**NLR**	17	Random	0.33	0.15–0.52	87.6%	0.000	3.55	0.000
**Country**								
China	6	Random	0.33	0.16–0.51	75.4%	0.001	3.72	0.000
Turkey	7	Random	0.46	0.01–0.90	90.4%	0.000	2.01	0.044
Other country	4	Random	0.09	−0.21 to 0.39	74.3%	0.009	0.58	0.564
**Type of depression**								
MDD	15	Random	0.308	0.104–0.512	87.8%	0.000	2.95	0.003
PSD	2	Fixed	0.51	0.36–0.67	36.3%	0.210	6.46	0.000
**Diagnostic criteria**								
DSM	16	Random	0.37	0.19–0.55	85.1%	0.000	3.96	0.000
ICD-10	1	/	−0.15	−0.31 to 0.01	/	/	1.79	0.073
**Matching age and sex simultaneously**								
Yes	14	Random	0.38	0.17–0.59	86.8%	0.000	3.58	0.000
No	3	Random	0.12	−0.24 to 0.48	86.9%	0.000	0.66	0.512
**Suicidal behavior**								
Yes	3	Random	1.34	−0.61 to 3.30	97.6%	0.000	1.35	0.178
No	3	Fixed	−0.07	−0.29 to 0.16	0.0%	0.557	0.58	0.563
**PLR**	10	Random	0.24	0.02–0.46	88.0%	0.000	2.15	0.031
**Country**								
China	6	Random	0.39	0.11–0.68	90.8%	0.000	2.70	0.007
Turkey	3	Random	−0.02	−0.44 to 0.41	80.5%	0.006	0.08	0.933
Spain	1	/	0.03	−0.28 to 0.34	/	/	0.17	0.863
**Type of depression**								
MDD	8	Random	0.16	−0.02 to 0.33	73.3%	0.000	1.75	0.080
PSD	2	Random	0.61	−0.30 to 1.52	97.0%	0.000	1.31	0.189
**Matching age and sex simultaneously**								
Yes	8	Random	0.17	0.01–0.33	72.4%	0.001	2.05	0.041
No	2	Random	0.56	−0.47 to 1.58	96.4%	0.000	1.06	0.289
**Suicidal behavior**								
Yes	2	Fixed	0.12	−0.15 to 0.40	0.0%	0.882	0.88	0.378
No	2	Fixed	−0.14	−0.41 to 0.12	0.0%	0.821	1.04	0.297
**MLR**	3	Random	0.15	−0.26 to 0.55	86.8%	0.000	0.71	0.475
**Country**								
China	2	Fixed	0.32	0.20–0.44	0.0%	0.401	5.16	0.000
Spain	1	/	−0.33	−0.64 to −0.02	/	/	2.07	0.038
**Type of depression**								
MDD	2	Random	0.00	−0.61 to 0.62	92.5%	0.000	0.01	0.994
PSD	1	/	0.46	0.12–0.79	/	/	2.65	0.008
**Matching age and sex simultaneously**								
Yes	2	Fixed	0.32	0.20–0.44	0.0%	0.401	5.16	0.000
No	1	/	−0.33	−0.64 to −0.02	/	/	2.07	0.038

*NLR neutrophil-to-lymphocyte ratio; PLR, platelet-to-lymphocyte ratio; MLR, monocyte-to-lymphocyte ratio; MDD, major depressive disorder; PSD, post-stroke depression; DSM, Diagnostic and Statistical Manual of Mental Disorders; ICD, International Classification of Diseases; SMD, standardized mean differences; 95% CI, 95% confidence interval; P_H_, p-value of heterogeneity; P, p-value*.

Meta-regression analyses showed that the differences in mean age and proportion of males in the control group were not the possible sources of heterogeneity. However, we observed a significant difference, namely that the male proportion in the case group influenced heterogeneity. The results of meta-regression are exhibited in Table 4. The funnel plot was roughly symmetric (Figure 2C), and Begg's and Egger's tests demonstrated no statistical difference (*p* = 1.000 and *p* = 0.165, respectively). These results indicated the absence of publication bias.

**Table 4:** Results of meta-regression analysis.

**Variables**	**Coefficients**	**Standard error**	**95% CI**	**z**	***p*-value**
**NLR**					
Age in case group	−0.005	0.009	−0.022 to 0.013	−0.56	0.583
Male proportion in case group	1.986	0.904	0.059–3.912	2.20	0.044
Age in control group	−0.006	0.009	−0.025 to 0.014	−0.62	0.547
Male proportion in control group	1.086	0.783	−0.583 to 2.755	1.39	0.186
**PLR**					
Age in case group	0.005	0.011	−0.021 to 0.031	0.48	0.646
Male proportion in case group	1.092	1.389	−2.111 to 4.295	0.79	0.454
Age in control group	0.005	0.012	−0.023 to 0.032	0.41	0.696
Male proportion in control group	1.338	0.922	−0.788 to 2.464	1.45	0.185

*NLR, neutrophil-to-lymphocyte ratio; PLR, platelet-to-lymphocyte ratio; 95% CI, 95% confidence interval*.

#### PLR

**Figure 3:**
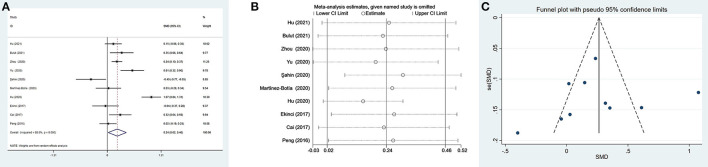
Results of meta-analysis on PLR between depressed patients and controls. **(A)** Forest plot of standardized mean difference in PLR between depressed patients and controls. The PLR values were distinctly higher in depressed patients than in controls (SMD = 0.24, 95% CI: 0.02–0.46, *p* < 0.05). Due to significant heterogeneity among studies (*I*^2^ = 88.0%, *p* < 0.001), the random-effects model was applied. **(B)** Sensitivity analysis indicated an unstable result of this meta-analysis. **(C)** The funnel plot appeared to be symmetric, which suggested no publication bias in studies reporting the values of PLR in depressed patients and controls.

Ten studies (17, 35, 37, 41, 42, 48, 49, 54–56) evaluated PLR between 1,399 patients with depression (534 males) and 1,727 controls (811 males). The PLR values were distinctly higher in depressed patients than in controls (SMD = 0.24, 95% CI: 0.02–0.46, *p* < 0.05), and the forest plot is shown in Figure 3A. Due to significant heterogeneity among studies (*I*^2^ = 88.0%, *p* < 0.001), the random-effects model was applied. Relevant sensitivity analysis after sequentially removing several studies displayed the lower and meaningless pooled SMD values (Figure 3B), meaning an unstable result of meta-analysis.

We performed four subgroup analyses depending on country, type of depression, matching in terms of age and gender, and suicidal behavior (Table 3). A significant difference between patients with depression and controls was found only in China, but not in Turkey and Spain. The PLR values of patients with depression were higher than controls in the subgroups of MDD and PSD, but no significant differences. Ten studies were divided into two subgroups according to whether participants were age and sex matched simultaneously. The results suggested that PLR were higher in depressed patients compared to controls only in the matched subgroup. Additionally, in spite of no statistical difference, depressed patients with suicidal behavior exhibited slightly elevated PLR values compared to controls. However, patients without suicidal behavior displayed slightly reduced PLR values.

As shown in Table 4, the meta-regression analysis revealed that age difference and male proportion were not the sources of heterogeneity. Visually, the funnel plot appeared to be symmetric (Figure 3C), and the results of Begg's test and Egger's test were not significant (*p*=0.283 and *p*=0.826, respectively). The results suggested no publication bias in reporting the values of PLR in depressed patients and controls.

#### MLR

**Figure 4:**
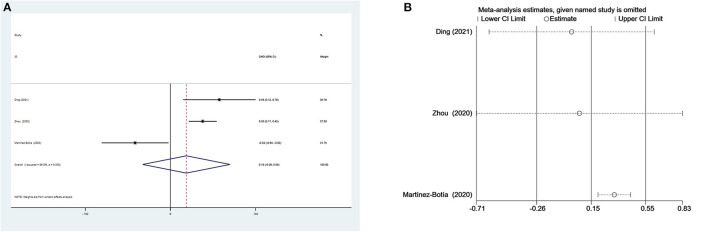
Results of meta-analysis on MLR between depressed patients and controls. **(A)** Forest plot Forest plot of standardized mean difference in MLR between depressed patients and controls. Depressed patients had slightly higher MLR than controls without a significant difference (SMD = 0.15, 95% CI: −0.26 to 0.55; *p* > 0.05). Heterogeneity among three studies remained high (*I*^2^ = 86.8%, *p* < 0.001) and the random-effects model was used. **(B)** Sensitivity analysis suggested that the results of the meta-analysis were unstable.

We could not acquire available MLR data from the study by Yu et al. (35) when comparing patients with depression and controls. Thus, only three studies including 569 individuals with depression and 710 controls were selected in the meta-analysis. Depressed patients had slightly higher MLR than controls without a significant difference (SMD = 0.15, 95% CI: −0.26 to 0.55; *p* > 0.05) (Figure 4A). Heterogeneity among the three studies remained high (*I*^2^ = 86.8%, *p* < 0.001) and the random-effects model was used. Sensitivity analysis eliminating one study (48) exhibited a higher and significant effect (SMD = 0.32, 95% CI: 0.20–0.44) (Figure 4B), which suggested that the results of the meta-analysis were unstable and that the study by Martínez-Botía et al. might be a source of heterogeneity.

There were three subgroup analyses for comparison of MLR values in depressed individuals and controls (Table 3). The same results of subgroup analyses based on country and matched age and gender revealed that MLR values were elevated in Chinese and age- and sex-matched depressed patients. In the subgroups of MDD and PSD, only patients with PSD had higher MLR values compared with controls. Meta-regression and publication bias analyses were not performed due to the small number of studies.

## Discussion

The updated meta-analysis including 18 studies suggested that higher peripheral blood inflammatory ratios, especially NLR, were significantly correlated with an increased susceptibility to depression. This result is consistent with that of the previous meta-analysis on MDD performed by Mazza et al. (45). But our meta-analysis provides more sufficient evidence to support the opinion that NLR may be a peripheral blood inflammatory biomarker of depression. Our findings may contribute to tailoring clinical decisions and improving the outcomes of patients with depressive disorders.

**Figure 5:**
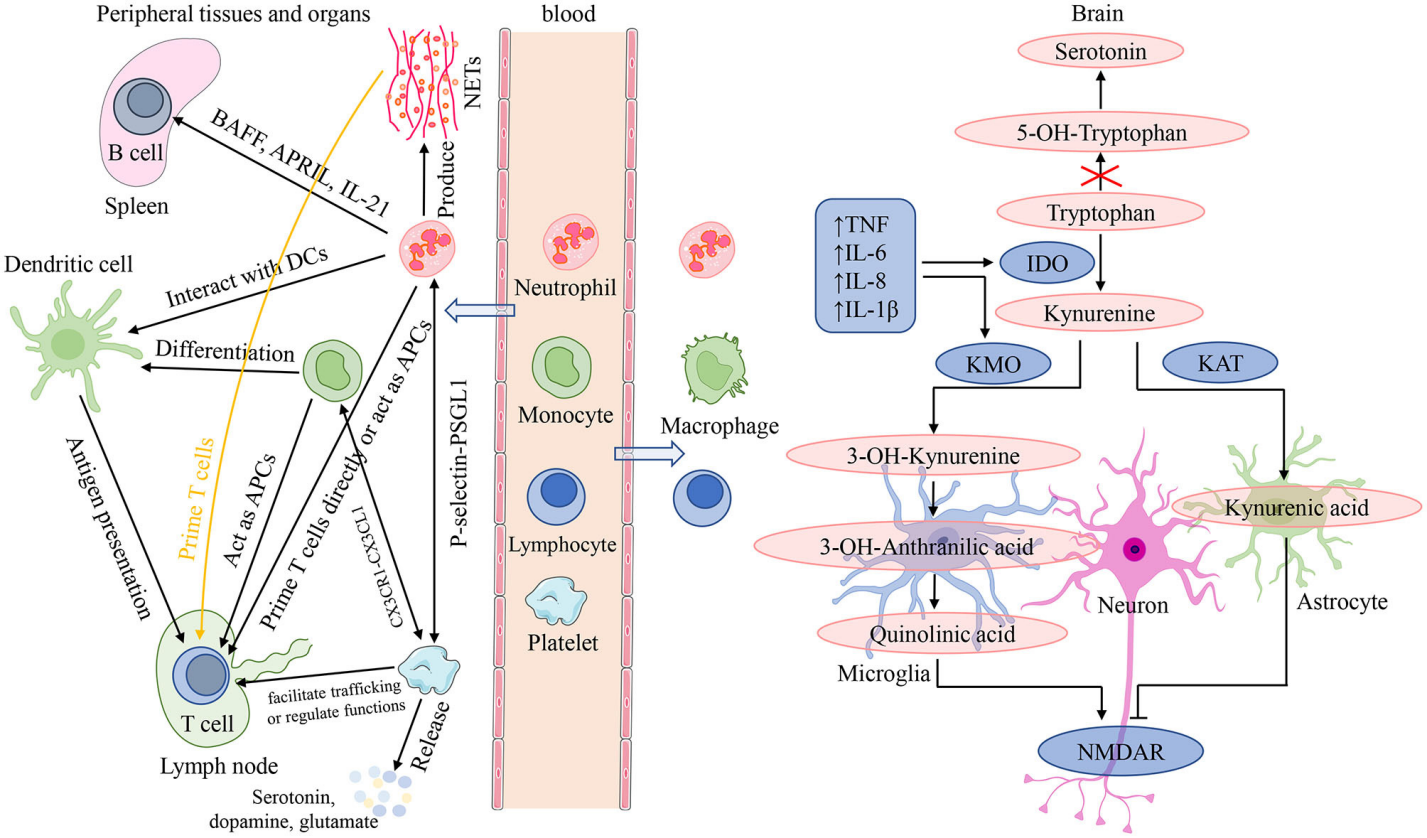
Interactions among peripheral blood cells and their involvement in the mechanisms of neuroinflammation induced depressive symptoms. In the presence of an inflammatory stimulus or infection, in addition to playing a role in recognizing, phagocytosis and killing pathogens, neutrophils can also interact with adaptive immunity. On the one hand, neutrophils can migrate to the spleen and induce T1 antibodies production by activating B cells through the productions of BAFF, APRIL and IL-21; while production of T1 antibodies helps to increase the phagocytic and clearance capabilities of neutrophils. On the other hand, neutrophils can facilitate the activation and differentiation of T cells through several following pathways: present antigens directly; by the release of NETs; acting as APCs; transfer antigens to dendritic cells. Monocytes can migrate into systemic tissue and differentiate into macrophages and dendritic cells, and play three major functions in immune responses: phagocytosis, antigen presentation, and cytokine production. For lymphocytes, they are the main effectors of adaptive immunity which facilitate pathogen-specific immune recognition, antibody, cytokine and immune memory production, cytotoxicity, and so on. Platelets are important effectors of innate and adaptive immunity: interact with neutrophils mediated by the P-selectin/PSGL1 axis; form the platelet-monocyte complexes through binding of platelet-expressed CXCR1 to CXCL1 of monocytes; facilitate T cell trafficking or regulate T cell functions. In addition, platelets can release serotonin, dopamine, glutamate, and so on. Chronic stress can disrupt the blood-brain barrier and increase access of peripheral cells to infiltrate directly into the brain. After migration into brain lesions, peripheral inflammatory cells (neutrophils, monocytes, and lymphocytes) may exacerbate brain injury and neuronal damage by releasing pro-inflammatory cytokines and NETs, and producing reactive oxygen species and reactive nitrogen species. neuroinflammation is orchestrated by resident immune cells such as microglia and astrocytes, as well as neutrophils, monocytes, and lymphocytes migrating from the periphery. It is able to influence depressive symptoms by impairing monoamine neurotransmitter synthesis, affecting glutamatergic signaling, and altering neurogenesis and synaptic plasticity. APC, antigen-presenting cell; APRIL, a proliferation-inducing ligand; BAFF, B cell activating factor; CXCL1, (C-X3-C Motif) Receptor 1; CXCRL, (C-X3-C Motif) Receptor 1; DC, dendritic cell; IDO, indoleamine 2,3-dioxygenase; IL, interleukin; KAT, kynurenine aminotransferase; KMO, kynurenine 3-monooxygenase; NETs, neutrophil extracellular traps; NMDAR, N-methyl-D-aspartate receptor; P-selectin, platelet selectin; PSGL1, P-selectin glycoprotein ligand 1; TNF, tumor necrosis factor.

Neutrophils are front-line troopers of innate immune defense, and engage in a variety of pathological and physiological processes such as injury, inflammation, autoimmunity and so on (57). Their core functions are to be recruited to the infection locus, recognize and phagocytose microorganisms, and then kill pathogens (58). Furthermore, neutrophils interact with adaptive immunity (Figure 5). Neutrophils activated by microbial pathogen-associated molecular patterns can migrate to the spleen and induce T1 antibodies production by activating marginal zone B cells through the productions of B cell activating factor, a proliferation-inducing ligand, and IL-21 (57). While production of T1 antibodies helps to increase the phagocytic and clearance capabilities of neutrophils. On the one hand, neutrophils can facilitate the activation and differentiation of T cells through several pathways (57, 59). First, neutrophils are capable of carrying antigens to migrate to lymph nodes and present them directly to T cells, or neutrophil extracellular traps (NETs) produced by neutrophils can prime T cells directly by lowering their activation threshold. Then, neutrophils also can act as antigen-presenting cells to present the antigen molecules to T cells, or can transfer antigens to dendritic cells and make dendritic cells more effective initiators of T cell activation, which facilitate TH1 and TH17 differentiation. On the other hand, neutrophils can inhibit the proliferation and activation of T cells, which probably attributes to the large increase in arginase 1 from neutrophil azurophilic granules and production of reactive oxygen species (57). Several studies have demonstrated that stress itself can disrupt the blood-brain barrier (BBB) increasing access of peripheral cells to infiltrate directly into the brain (60–62). Neutrophils are able to migrate to the central nervous system to exacerbate neuroinflammation (63). After migration into brain lesions, neutrophils may exacerbate brain injury and neuronal damage by releasing pro-inflammatory cytokines and NETs, and producing reactive oxygen species and reactive nitrogen species (Figure 5). On the contrary, a neuroprotective effect of N2 neutrophils in post-ischemic inflammation was reported by several studies (63).

For lymphocytes, they are the main effectors of adaptive immunity which facilitate pathogen-specific immune recognition, immune memory production, and regulation of host immune homeostasis (64) (Figure 5). Lymphocytes perform a regulatory or protective function in adaptive immunity and reduced lymphocytes indicate poor general condition and physiological stress (20, 45). In a review, Leite et al. (63) elaborated that neuroinflammation is orchestrated by resident immune cells such as microglia and astrocytes, as well as lymphocytes migrating from the periphery. An immunohistochemical study from post-mortem brain samples suggested that a higher percentage of patients with schizophrenia and mood disorders suffered from lymphocyte infiltration compared to healthy controls (65). And elevated lymphocyte density in brain regions is recognized as an indicator of neuroinflammation associated with the impaired BBB. Lymphocytes migrating into brain tissue, along with other peripheral cells and pro-inflammatory cytokines, serve as disease regulators in genetically susceptible individuals, triggering exacerbation of psychiatric or mood symptoms by aggravating the pre-existing disturbances in serotonergic, dopaminergic, and glutamatergic neurotransmission (66, 67) (Figure 5).

NLR is a marker of systemic inflammation and reflects two different immune pathways. It was first applied in critical medical patients to supervise the intensity of stress and/or systemic inflammation (19). Over time, the interest in it has grown, and it is investigated in multiple diseases. Our study, with a sufficient number of articles, demonstrates that NLR is higher in patients with depression compared to controls, which is consistent with that of the previous meta-analysis. This result seems to indicate that innate immunity, rather than adaptive immunity, is more responsible for the pathogenesis of depression. Our speculation is in accordance with a review showing that innate immunity dysregulation and uncontrolled neuroinflammatory processes appear to be critical in depression (68). After transfusion of lymph node cell suspensions from chronic stress mice in Rag2 knockout mice without mature lymphocytes, recipients developed an anti-inflammatory like responses in contrast to the apparent pro-inflammatory profile in donors (69). This preclinical study suggests that adaptive immune cells are probably reprogrammed by stress to some degree to promote resilience. Furthermore, one recent clinical study revealed increased neutrophils and CD4^+^ T cells (not all lymphocytes) in peripheral blood from MDD patients (70). Previous investigations have also observed neutrophils, without lymphocytes, were elevated in animals suffering from prolonged periods of stress and in major depressed patients (71, 72). These studies all support this opinion that innate immunity is more responsible for the pathogenesis of depression, compared to adaptive immunity.

Stratifying the studies for diagnosis, we discovered elevated NLR values in both subgroups. Interestingly, although only two studies were included, we noticed that NLR were high in PSD patients compared to non-PSD patients. PSD is a frequent neuropsychiatric disorder, impacting rehabilitation, recuperation of motor and cognitive deficits following stroke, and dramatically increasing the risk of stroke recurrence and mortality (73, 104). A meta-analysis including 61 cohorts covered that the pooled morbidity of PSD amounted to 31% for 5 years post-stroke (74). Clinical studies and animal experiments have confirmed that inflammation is associated with PSD (75–77). Moreover, increased NLR was discovered to correlate with the occurrence of PSD (41, 54, 78). This result of subgroup analysis on PSD, excluding one from three studies due to overlapped participants, suggests that an elevated NLR value in the acute phase of stroke seems to be a reminder of the increased susceptibility to PSD. This speculation demands further studies to be validated. In the subgroup according to DSM, depressed patients had obvious higher NLR values than controls. However, the subgroup analysis based on the ICD-10 had a conflicting result, namely that the NLR value was low in depressed patients without a significant difference. Due to only one study being included in this subgroup, more clinical research is required. Significant differences in NLR values among cases and controls were identified in China and Turkey, and even more so in China. However, in the subgroup comprised of Spain, the USA, Poland, and Germany, depressed patients did not show significantly higher NLR values than controls. This may be owing to the fact that the number of studies in this subgroup is low, only four, as well as the geographical differences between countries.

In addition, we discovered that NLR was significantly elevated in the subgroup of matched age and sex but was not in the unmatched subgroup. Moreover, the meta-regression analysis revealed a significant difference, being that the male proportion in the case group affected heterogeneity. These findings seem to imply that sex may have an impact on NLR levels in depressed patients. Women are nearly twice as likely as men to suffer from depression, and this female predilection occurs in adolescence and stays stable during adulthood (79). Current studies surrounding whether sex differences may influence the association of peripheral inflammatory markers with depression are sparse and controversial. Birur et al. (80) found that depressed women had higher pro-inflammatory factor, such as IFN-γ and IL-8, and lower levels of the anti-inflammatory adiponectin and the pro-inflammatory IL-5 compared to healthy women, but there are no equivalent findings in depressed men. Additionally, another study reported that higher serum C-reactive protein levels correlated with greater depression severity in female depressed patients, while there was no such correlation in males with depression (81). However, these findings are in contrast with the observation from several studies, namely that an association between C-reactive proteins and depression in men but not in women (82–84, 101). Despite the inconsistency of these findings, they support, to some extent, our speculation that gender differences do have an effect on NLR values in individuals with depression. Unfortunately, we did not perform the subgroup analysis according to sex differences since women and men with depression were not grouped in most of the included studies. Suicide is the primary cause of non-natural death globally and the most devastating outcome for people with depression. Major depressive disorder (MDD) is the most prevalent psychological disorder among individuals with suicidal behavior, and patients with depression had a 20-fold suicide mortality compared to the general population (48). A review by Brundin et al. (85) suggests that inflammation may be especially pronounced in patients with suicidal behavior. More recently, Velasco et al. (43) identified NLR as a potential new peripheral biomarker of suicidal behavior in MDD. Although only three studies were selected for the subgroup analysis based on whether there was a suicidal behavior, we observed that depressed patients who experience suicidality were associated with increasing NLR value. The finding that depressed patients without suicidal behavior showed slightly lower NLR values than controls is inconsistent with the overall result of NLR and depression. Further investigations are urgently required to confirm the above findings, owing to the small number of included studies and no significant results in this subgroup analysis.

PLR is another peripheral marker of systemic inflammation. Platelets are prevalent non-nucleated blood components and the primary cell regulating hemostasis and thrombosis (86). Several recent studies have shown that platelets are important effectors of innate and adaptive immunity (86–88) (Figure 5). In the setting of Toll-like receptor agonists and viral infections, platelets interact with neutrophils mediated by the platelet selectin/platelet selectin glycoprotein ligand 1 axis to form heterotypic aggregates and initiate innate immune responses (86). In addition, platelet interaction with neutrophils contributes to NET formation termed netosis. In the presence of inflammatory stimuli or infection, platelets are able to interact with monocytes, and Postea et al. (89) found that the platelet-monocyte complexes can be formed through binding of the platelet-expressed chemokine (C-X3-C Motif) Receptor 1 to the (C-X3-C Motif) ligand 1 of monocytes (add). When platelets and B-cells were co-incubated *in vitro*, platelet content could contribute to production of IgG (IgG1, IgG2, and IgG3) from B cells (90). For T cells, it was found that platelets may facilitate T cell trafficking to the sites of inflammation or infection through platelet interactions with lymphocytes (86). Furthermore, activated platelets may regulate T cell functions through release of some factors such as serotonin or platelet factor 4 (86). Alongside with acting as an immune effector, platelets share several characteristics with neurons (Figure 5). They all have similar secretory vesicles with regard to content, storing molecules such as serotonin or 5-hydroxytryptamine (5-HT), dopamine, glutamate, gamma-aminobutyric acid, and so on (91). Furthermore, they also share several proteins, including serotonin transporters (SRET) and receptors (e.g., serotonin receptor 2A and 3A), as well as a number of neuron-associated markers, such as brain-derived neurotrophic factor (91). These shared contents and proteins have an essential role in the pathophysiology of psychotic disorders and are the targets of antipsychotic treatment. One study suggested that platelet parameters, platelet count (PLT), mean platelet volume, and platelet large cell ratio were significantly related to psychiatric disorders (92).

As the ratio of platelets to lymphocytes, PLR can also serve as a predictor of inflammatory response, but seems to be less effective than NLR. The results of our meta-analysis on PLR validate this hypothesis. Although the overall results showed that depressed patients had significantly higher NLR values than controls, the relevant sensitivity analysis showed that the results were unstable. Meanwhile, subgroup analysis also revealed significant differences among depressed patients with controls only in China and in the age- and sex-matched subgroup. In our opinion, apart from regional differences and heterogeneity among studies, the small function of PLR values themselves on depression is also an important reason for the results of this meta-analysis. Platelets are weaker indicators than neutrophils in terms of inflammation, and the evidence (91) for the relationship between PLT, as one of the platelet parameters mimicking neuronal characteristics, and MDD is conflicting, which may be the reasons for the small function of PLR values. As with NLR values, depressed patients with suicidal behavior were associated with increasing PLR value in a subgroup analysis including two studies. Meanwhile, the PLR values were similarly slightly lower in patients without suicidality than in the control group. Combined with the subgroup analysis of NLR based on the presence or absence of suicidal behavior, our findings support this argument that inflammation may be particularly pronounced in patients with suicidal behavior. But whether there is absence of inflammatory response for depressed individuals without suicidality warrants more research to determine. This is the first time that more than four studies have been included in a meta-analysis to explore the role of PLR in depression. Considering the relatively few included studies and some limitations in our meta-analysis, it will be appropriate for further research on this role to be conducted.

Monocytes from bone marrow are released into the bloodstream to circulate for 1–3 days, and then they migrate into systemic tissue and differentiate into macrophages and dendritic cells (93). Monocytes and their differentiated cells have three major functions in immune responses: phagocytosis, antigen presentation, and production of cytokines such as IL-1, IL-6, IL-8 and TNF-α (93, 94) (Figure 5). In innate immunity, monocytes can migrate to infected tissues or lymph nodes and then differentiate into macrophages that phagocytose microorganisms and scavenge cellular debris, as well as mature into TNF- and inducible NO synthase (iNOS)-producing dendritic cells with the size and characteristics of monocytes (95). While in adaptive immunity, monocytes can act as antigen-presenting cells to acquire antigens in peripheral tissues and conserve and process antigenic peptides. After migrating into lymph nodes, monocytes present antigen to naive T-cell, promoting their activation, clonal expansion, and differentiation (95). Clinical studies have shown that peripheral blood monocytes were elevated in MDD patients (70, 72). Meanwhile, animal research has also reported elevated monocytes following prolonged periods of stress (71). Except for elevated monocytes in peripheral blood, repeated stress exposure facilitates the entrance of peripheral monocytes into the brain where they cooperate with microglia, contributing to development of neuroinflammation and anxiety-like behavior (20, 96–98). Neuroinflammation is also able to influence depressive symptoms by impairing monoamine neurotransmitter synthesis, affecting glutamatergic signaling, and altering neurogenesis and synaptic plasticity (5, 99) (Figure 5). A study performed on post-mortem brains showed that a greater number of peripheral monocytes were really recruited to the brain of depressed suicidal patients than controls (12). Subsequent research from animal models found that genetic and pharmacological approaches, blocking the pathway of monocytes into the brain and reducing the production of pro-inflammatory cytokines, could decrease anxiety- and depressive-like behaviors (5, 100).

Taking monocytes with lymphocytes together, we considered that MLR, as a peripheral marker, could suggest neuroinflammation in patients with depression. In our meta-analysis, patients with depression showed a trend of increased MLR than controls. Interestingly, a higher and significant effect was found when leaving out one study (48) in sensitivity analysis. Moreover, the subgroup analyses based on country and matched age and gender yielded the same results as the sensitivity analysis. Taken together, these results supported the view that MLR might be a peripheral marker of neuroinflammation in depressed patients and suggested that the study by Martínez-Botía et al. (48) might be responsible for the heterogeneity. In addition, one study measured MLR in PSD, observing higher MLR value in PSD patients compared to non-PSD patients. Nevertheless, less is known about what role this marker serves in depression and PSD, and further research is deserved.

It should be acknowledged that there are the following inherent limitations in our study. First, heterogeneity across studies was high, which partially limits the generalizability of our results. This probably indicated that there were discrepancies among studies in the field of recruiting backgrounds, inclusion criteria, assessments of depression, sex differences in depressed patients, characteristics of controls, and type and quality of studies. Second, despite the finding that sex differences are a reason for high heterogeneity across studies assessing NLR values, we didn't conduct a subgroup analysis based on sex differences due to limitations in the studies themselves. Third, some covariates that might impact the complex associations involving inflammatory ratios and depression, such as treatment status, recruitment settings, lifestyle, smoking, and body mass index, were not specifically analyzed. Fourth, the paucity of relevant data in studies prevented us from evaluating the correlation of depression severity with inflammation ratios. Fifth, evidence to determine whether these inflammatory ratios delegate independent peripheral markers of immune system activation in patients with depression is insufficient since other mediators associated with immune response, such as IL-6, IL-1β, TNF, and C-reactive proteins, were not measured. Finally, the quantity of studies accepted for the meta-analysis on MLR is comparatively little, which reduces the credibility of corresponding results.

## Conclusions

In summary, our findings are significant and contribute to the field. Compared to the previous meta-analysis, more than 3-fold studies with above 4,000 individuals were added, and more categories of analysis were performed, generating some more compelling and interesting results. In this newly updated meta-analysis, higher NLR and PLR, especially NLR, were still significantly associated with an increased risk for depression. In the subgroup of China and matched age and gender, NLR, PLR, and MLR were all elevated in depressed patients vs. controls. Interestingly, NLR and MLR were both measurably increased in PSD patients. In addition, we speculate that gender differences may have an effect on NLR values in individuals with depression. Finally, we observed that elevated NLR and PLR might be particularly pronounced in depressed patients with suicidal behavior.

## Data Availability Statement

All datasets generated for this study are included in the article/Supplementary Material.

## Author Contributions

SF and LZ conceived and directed this study. YC, YW, XW, and ZJ contributed to literature search, data extraction, and data analysis. YC and YW were responsible for the results verification of meta-analysis. YC was responsible for manuscript writing. SF, LZ, and YC contributed to writing-review and revising the manuscript. All authors have read and approved the final version of the manuscript and agreed to submit it for publication.

## Funding

The study was supported by grants from the National Natural Science Foundation of China (grant number 81873794).

## Conflict of Interest

The authors declare that the research was conducted in the absence of any commercial or financial relationships that could be construed as a potential conflict of interest.

## Publisher's Note

All claims expressed in this article are solely those of the authors and do not necessarily represent those of their affiliated organizations, or those of the publisher, the editors and the reviewers. Any product that may be evaluated in this article, or claim that may be made by its manufacturer, is not guaranteed or endorsed by the publisher.
