# Supplementary material for: Neutrophil-to-Lymphocyte Ratio, Platelet-to-Lymphocyte Ratio, and Monocyte-to-Lymphocyte Ratio in Depression: An Updated Systematic Review and Meta-Analysis
Source: Front Psychiatry. 2022 Jun 15;13:893097. doi: 10.3389/fpsyt.2022.893097 (PMC9240476; doi:10.3389/fpsyt.2022.893097)
Supplement: Supplementary file 1 [file Table_1.docx]

**TABLE s1 |** Newcastle–Ottawa Quality Assessment Scale for Cohort Studies, Case–Control and Cross-Sectional Studies.

| Cohort studies (n=2) | Selection | | | | Comparability | Outcome | | | Score |
| --- | --- | --- | --- | --- | --- | --- | --- | --- | --- |
|  | Representativeness of the exposed cohort | Selection of the non-exposed cohort | Ascertainment of exposure | Outcome of interest was not present at start of study | Comparability of cohorts on the basis of the design or analysis | Assessment of outcome | Was follow-up long enough for outcomes to occur | Adequacy of follow-up of cohorts |  |
| Hu et al., 2021 | 0 | 1 | 1 | 1 | 2 | 1 | 1 | 1 | 8 |
| Ding et al., 2021 | 0 | 1 | 1 | 1 | 2 | 1 | 1 | 1 | 8 |
| Hu et al., 2020 | 0 | 1 | 1 | 1 | 1 | 1 | 1 | 1 | 7 |
| Case-Control and Cross- Sectional Studies (n=16) | Selection | | | | Comparability | Exposure | | | |
|  | Is the case definition adequate? | Representativeness of the cases | Selection of controls | Definition of controls | Comparability of cases and controls on the basis of the design or analysis | Assessment of exposure | Same method of ascertainment for cases and controls | non-response rate | Total score |
| Bulut, et al., 2021 | 1 | 1 | 0 | 1 | 2 | 1 | 1 | 0 | 7 |
| Zhou et al., 2020 | 1 | 1 | 0 | 1 | 2 | 1 | 1 | 0 | 7 |
| Yu et al., 2020 | 0 | 1 | 0 | 1 | 2 | 1 | 1 | 0 | 6 |
| Şahin et al., 2020 | 1 | 0 | 0 | 1 | 2 | 1 | 1 | 0 | 6 |
| Martínez-Botía et al., 2020 | 0 | 1 | 0 | 1 | 1 | 1 | 1 | 0 | 5 |
| Grudet et al., 2020 | 1 | 1 | 1 | 1 | 2 | 1 | 1 | 0 | 8 |
| Öztürk et al., 2019 | 0 | 1 | 0 | 1 | 2 | 1 | 1 | 0 | 6 |
| Arabska et al., 2018 | 0 | 1 | 0 | 1 | 1 | 1 | 1 | 0 | 5 |
| Euteneuer et al., 2017 | 0 | 1 | 1 | 1 | 2 | 1 | 1 | 0 | 7 |
| Ekinci et al., 2017 | 1 | 1 | 0 | 1 | 2 | 1 | 1 | 0 | 7 |
| Cai et al., 2017 | 1 | 1 | 1 | 1 | 2 | 1 | 1 | 0 | 8 |
| Peng et al., 2016 | 1 | 1 | 0 | 1 | 2 | 1 | 1 | 0 | 7 |
| Korkmaz et al., 2016 | 0 | 1 | 0 | 1 | 2 | 1 | 1 | 0 | 6 |
| Demircan et al., 2016 | 1 | 1 | 0 | 1 | 2 | 1 | 1 | 0 | 7 |
| Demir et al., 2015 | 0 | 1 | 0 | 1 | 2 | 1 | 1 | 0 | 6 |
